# The Role of Translation in Citizen Science to Foster Social Innovation

**DOI:** 10.3389/fsoc.2021.629720

**Published:** 2021-03-24

**Authors:** Barbara Heinisch

**Affiliations:** Centre for Translation Studies, University of Vienna, Vienna, WI, Austria

**Keywords:** translation, localization, adaptation, social change, terminology

## Abstract

Citizen science has become a world-wide phenomenon. Especially for citizen science projects that have a global reach, translation is crucial to overcome language and cultural barriers to reach members of the public. Translation, understood as the transfer of meaning (of a text) from one language into another language, is crucial for the transmission of information, knowledge and (social) innovations. Therefore, this paper examines the role of translation and terminology used in citizen science projects and how translation can foster (or impede) social innovation through citizen science activities. Based on a set of predefined criteria derived from the social innovation literature, this paper analyzes the factors that contribute to (social) innovation in citizen science by means of translation. A specific focus of the case study is on the aspects of agency, institutions, and social systems. The results demonstrate that translation in citizen science may support a change of social practices as ingredients of social innovations. Additional research is needed to further understand the implications of translation in citizen science and its effects on social innovation. Nevertheless, this work has been one of the first attempts to examine the relation between translation, citizen science and social innovation.

## Introduction

Citizen science has received considerable attention in recent years. Although citizen science has been practiced for a long time, it evolved as a “movement” only recently. “Citizen science projects actively involve citizens in scientific endeavor that generates new knowledge or understanding. Citizens may act as contributors, collaborators, or as project leader and have a meaningful role in the project” (European Citizen Science Association, 2015). The increased interest in and emergence of citizen science led to a professionalization of the field, the development of a community (of practice) and of principles of citizen science (European Citizen Science Association, 2015; ECSA, 2020). These principles specify inclusion and exclusion criteria to draw boundaries between what can, and cannot be considered citizen science.

The different ways how citizen science is understood have led to new forms of engaging with the public, including aspects of diversity, creativity and social innovation (Schäfer and Kieslinger, 2016). Moreover, aspects such as ethics, transparency, recruitment of participants, including citizen science project platforms, easily understandable data protocols and communication of results to the public as well as co-authorship of citizen scientists in academic publications receive considerable attention in the literature.

### Translation and Innovation

However, much less is known about the role of translation in citizen science. Traditionally, translation is defined as the transfer (of meaning) of a text from a source language into a (text in the) target language (Snell-Hornby, 2005). This shows that at the heart of this transfer are not words or languages, but texts. Translation is crucial for all fields of human activity, ranging from governance and economy to culture and literature (Woodsworth, 2013) and it enables communication and understanding between different language communities (Burnett, 2018).

When referring to the translation of citizen science projects or translation for citizen science projects, it must be considered that the texts to be translated are embedded in a context, i.e., a situation characterized by historical, cultural and socio-economic aspects. Translation is thus a form of transcultural communication, a communication determined by a certain purpose and targeted at a certain audience. This purpose (skopos) influences the realization of the translation, including its content, form, style, etc. (Vermeer, 1978).

Translation practice has undergone several changes and translation theory has seen various paradigm shifts. Among the major impacts (or innovations) are technology and crowdsourced translation.

Technological advances, including computer-assisted translation (CAT) tools and machine translation changed the way translators work. CAT tools designate a software that assists translators while translating. They facilitate the translation process by increasing the speed and the quality of the translation. At the heart of a CAT tool is a translation memory, i.e., a database that stores pairs of translation units, usually sentences, in the source and target language that were previously already translated. If the same or similar sentences occur in the text to be translated, the software displays these to the translator (Braun, 2015). CAT tools have therefore drastically impacted the translation process (Christensen and Schjoldager, 2010) since translators now work in a segment-by-segment manner. The positive effects are that it increases productivity and consistency. The negative effects are related to text cohesion and overreliance on translation units suggested by the translation memory (regardless of their quality) (Krüger, 2016). Another technology that impacted the translation process is machine translation. Especially neural machine translation has made major progress in recent years and reaches good quality in various language pairs. Machine translation systems are not only used by professional translators to increase productivity but also by various other user groups, also due to freely available machine translation systems on the Internet. Since both CAT tools and machine translation systems have revolutionized translation processes, they are also helpful tools in the case of crowdsourced translation.

Crowdsourced translation (also partly referred to as community translation or volunteered translation) refers to “translation where the members of the undefined “crowd” act as volunteer translators” (O’Hagan, 2012). Here, “the Internet provides a platform for completing tasks relying on the knowledge of a self-selected community of volunteers on the web” (Jiménez-Crespo, 2013). The Internet and technological advances allow for user participation and online collaboration among large user groups. Crowdsourced translation ranges from the translation of popular culture, including fansubbing, where fans create the subtitles of films or TV programs in another language, to the translation of social media platforms by their users, such as Facebook, or subtitling of TED talks by volunteers (O’Hagan, 2012). Citizen science initiatives also make use of crowdsourced translation, such as the Citizen Science Translation Hub (citscitranslate.wixsite.com/citscitranslate), where crowdsourced translation meets citizen science: “Help us out as a volunteer or proofreader. No experience required, just the ability to speak more than one language!”

Both translation technologies and the crowd help to address emerging translation needs. Both should increase productivity and accelerate the translation of large volumes of text (and reduce costs of translation) (Anastasiou and Gupta, 2011).

However, very little is currently known about the relation between translation and social innovation in citizen science.

### Social Innovation

Similar to citizen science, social innovation is a concept that still lacks a uniform definition among the research community. However, in this paper the definition by Howaldt and Schwarz (2010) is used: “A social innovation is new combination and/or new configuration of social practices in certain areas of action or social contexts prompted by certain actors or constellations of actors in an intentional targeted manner with the goal of better satisfying or answering needs and problems than is possible on the basis of established practices”.

Social innovation thus results in new solutions, such as products, processes, activities or services that satisfy a social need and enhance a society’s capacity to act. Social innovation depends on the contribution and participation of all actors (Portales, 2019). In contrast to other forms of innovation, social innovation is not aimed at maximizing profit and having a competitive advantage but is driven by the concern for communities (a social need or social problem) and results in social change among a large number of people (do Adro and Fernandes, 2020).

Three agents in social innovation have been proposed, namely individuals, organizations and social movements. Although there are also other agents, such as governments and enterprises, these can only coordinate (do Adro and Fernandes, 2020).

Therefore, social innovation depends on agents, on the one hand, and (social) structures, on the other. This means that social innovation is created by agents, i.e., actions or behaviors by individuals (that result in collective actions within a social system) and the external structural context (since a social system is characterized by its underlying institutions). Social innovation, thus, requires action and the reproduction of these actions (Cajaiba-Santana, 2014).

Therefore, social innovation is characterized by innovation, agents, structures or institutions and a social system. Thus, the relationship between actors and structures is key to social innovation (Cajaiba-Santana, 2014). The change that results from social innovation targets social practices. It manifests in “changes in attitudes, behaviors, or perceptions, resulting in new social practices, new institutions, and new social systems that allow visualizing a real transformation of society” (Portales, 2019). Social innovations are rooted in their social context defined by various historical and cultural framework conditions. Therefore, actions and the social context are intertwined (Cajaiba-Santana, 2014).

Social innovators foster social transformation. They can be any actor, i.e., individuals or entire communities independent of the sector of society. However, social change can only be achieved if actors from all sectors participate in social innovation processes, since critical actors are crucial to solve a complex problem (Portales, 2019).

Moreover, another important differentiation in social innovation is the difference between result and process. Regarding the result, social innovation emphasizes the satisfaction of a certain need through innovation, as well as generating new social structures and improved relationships in society. From a long-term perspective, social innovation should increase a society’s capacity to act, by being aimed at a systemic societal transformation. Regarding the process, on the other hand, social innovation is a participatory process that enhances the relationship between actors, fostering social resilience and providing access to resources to meet certain needs (also) in the future (Portales, 2019).

### Citizen Science as Social Innovation and Citizen Science Resulting in Social Innovation

Citizen science itself results in a change of social practices. Therefore, citizen science can be regarded as social innovation (Butkevičienė et al., 2021). Therefore, we can observe an effect of citizen science (practice) on academia that allows to classify citizen science as social innovation in scholarship. Social innovation and citizen science share many commonalities. Both are cutting-edge, embrace (technological) advances and social objectives. However, it still needs to be investigated if citizen science can produce long-term change in academia and thus also transform social systems.

Although citizen science has received special attention recently, also in (European) funding schemes, the recognition of researchers engaging in citizen science, as well as academic incentives for citizen science activities are lagging behind, such as a proposed social impact indicator (Schäfer and Kieslinger, 2016). This is despite the fact that citizen science can open up academia, which is often characterized as ivory tower, detached from the world “outside”, disconnected from reality and practical considerations. However, citizen science is praised as democratization of research (Irwin, 1995) and a means to raise awareness for and knowledge of certain topics, to increase scientific literacy (Bonney et al., 2009; Queiruga-Dios et al., 2020), to change attitudes (Brossard et al., 2005) and tackle societal problems (Dickinson et al., 2013). Moreover, citizen science can result in the empowerment of the participants (Socientize, 2013; Göbel et al., 2019), similar to participatory action research. However, citizen science also faces challenges ranging from data quality considerations (See et al., 2013) to ethical issues such as the exploitation of free labor, or inclusion.

### Translation, Citizen Science and Social Innovation

Citizen science has proliferated in recent years, as does social innovation. Citizen science has been framed as social innovation itself, and it can also be the basis for social innovation and, thus, social change. Translation has also been characterized as a means to foster (or impede) change in societies and cultures.

No previous study has investigated the interplay between citizen science, translation and social innovation. Therefore, this study examines the role of translation used in citizen science projects and addresses the question of how translation can foster (or impede) social innovation through citizen science activities.

While some research has been carried out on translation in citizen science projects (Michalak, 2015; Desjardins, 2021) and a Citizen Science Translation Hub was launched (Sheppard, 2020), there is still very little academic understanding of transcultural issues of citizen science and social innovation, especially with regard to the aspect of translation. Based on a case study, this paper explores the ways in which translation is used to meet the needs of the contributing participants and the extent to which translation in citizen science projects can bring social innovation. Understanding the link between translation in citizen science projects and social innovation will help consider these aspects in citizen science in the future.

## Methods and Materials

This paper examines the role of translation and terminology used in citizen science projects and how translation can foster (or impede) social innovation through citizen science activities.

Based on a set of predefined criteria derived from the social innovation literature with regard to the key dimensions and characteristics of social innovation, this paper analyzes the factors that contribute to (social) innovation in and through citizen science by means of translation. A specific focus is the change of social practices fostered by translation in citizen science activities and the underlying aspects of agency, institutions, and social systems.

A case-study approach was adopted to allow a deeper insight into the translation aspect in citizen science projects that have a global reach. The projects for the study were selected from the citizen science project platform Zooniverse based on their international nature, the availability of the Zooniverse (project) pages in at least two languages, including English, and the consideration of localization.

To examine the role of translation in citizen science projects to foster (or impede) social innovation exemplified by Zooniverse, this study further explores the languages represented in Zooniverse projects, the way how translation is dealt with on Zooniverse and the features of social innovation reflected in translation.

For this purpose, the Zooniverse website was analyzed, in particular the multilingual project pages (active projects under zooniverse.org/projects), the general forums (zooniverse.org/talk), the blog (blog.zooniverse.org) as well as the help page (help.zooniverse.org). A list of all active project pages available in more than one language was compiled, including the languages in which these project pages are available. The forums and the blog were searched by using the following keywords: “translat*” (to include translation, translated, translate, translating, etc.), “locali*” (to include localization, localized, localizing, etc.), “adapt*” (to include adaptation, adapting, adapted, etc.) and “language”. Forum posts that contained these key words but were not relevant to the current research were excluded from the further analysis. The criteria used for analysis are described in the following.

### Criteria of Analysis

The social innovation literature specifies various characteristics, principles and methods of analysis of social innovation (Table 1). While, from a process perspective, agency, institutions and social systems can be differentiated (Cajaiba-Santana, 2014), from a dimension perspective, concepts and understanding, objectives (societal challenges, systemic changes), drivers and barriers (including governance), the social innovation cycle and resources, capabilities, and constraints (including finance, regulations, human resources, and empowerment) can be identified (Howaldt et al., 2014).

**TABLE 1:** List of social innovation aspects (non-exhaustive).

Aspect	Criteria
Process perspective Cajaiba Santana (2014)	Agency
	Institutions
	Social systems
Three dimensions of social innovation Moulaert et al. (2005)	Content (satisfaction of human needs)
	Process (changes in social relations)
	Empowerment (increase in socio-political capability and access to resources)
Four key elements of social innovation Portales (2019)	Satisfaction of a need
	Innovation of the solution
	Change of social structures and relationships
	The increase in society’s capacity to act
Key dimensions of social innovation Howaldt et al. (2014)	Concepts and understanding
	Addressed societal needs and challenges
	Resources, capabilities and constraints
	Process dynamics
	Actors, networks and governance
Engaged research Stanton (2008)	Purpose
	Process
	Product
Ten social innovation influencing factors Oganisjana et al. (2015)	Openness to novelty
	Consciousness
	Responsibility
	Proactive thinking
	Lifelong learning
	Positive experience
	Passivity
	Conservative thinking
	Power distance
	Bureaucracy
Four layers of social innovation ecosystems Kaletka et al. (2016)	Context of roles
	Context of functions
	Context of structures
	Context of norms
Five main definitions for the concept of social innovation that leads to social change Tardif and Harrison (2005)	Novelty and character of innovation
	Objective of innovation
	Innovation process
	Relationship between actors and structures
	Restrictions on innovation
Five dimensions of social innovation Tardif and Harrison (2005)	Transformation
	Innovative character
	Innovation
	Actors
	Processes
Levels of analysis and occurrence of social innovation Cajaiba-Santana (2014)	Intra-social group innovations
	Inter-social group innovation
	Extra-group social innovations

Additionally, ten factors that have an influence on social innovation are specified: openness to novelty, consciousness, responsibility, proactive thinking, lifelong learning, positive experience, passivity, conservative thinking, power distance and bureaucracy (Oganisjana et al., 2015). Focus group discussions revealed that the six categories *openness to novelty, proactive thinking, consciousness, responsibility, lifelong learning, and positive experience* can promote social innovation if they are present (and hinder social innovation if they are absent). The other four categories (*conservative thinking, passivity, power distance, and bureaucracy*) were identified as factors clearly hindering social innovation (Oganisjana et al., 2015).

Despite the different approaches to the concept of social innovation, it has several key elements. The number of those differs between authors. Social innovation has three key elements according to Moulaert et al. (2005): First, the content or product dimension, which consists of the satisfaction of a (yet unsatisfied) human need. Second, the process dimension, referring to changes in social relations, especially governance and the participation of disadvantaged groups. Third, the empowerment dimension, consisting of the increase in the socio-political capability and the access to resources. An additional key element identified by Portales (2019) is the innovation aspect, thus, resulting in these four key elements of social innovation: the satisfaction of a need, the innovation of the solution, the change of social structures and relationships and the gain of a society’s capacity to act.

The criteria of content, process and empowerment (impact) were also used to analyze social innovation with respect to citizen science (Butkevičienė et al., 2021). From the perspective of engaged research, purpose, process and product may be criteria for analysis (Stanton, 2008).

Other aspects identified that help social innovation to achieve social change (according to Tardif and Harrison (2005) cited in Agostini et al. (2017)) are: the novelty and the nature of innovation, the objective of innovation, the innovation process, the relationship between structures and actors and innovation restrictions. Therefore, they suggested five dimensions of social innovation: transformation, innovative nature, innovation, processes and actors.

The model of the four layers of social innovation ecosystems (Kaletka et al., 2016) on the other hand, aims at understanding the complexity of the emergence of social innovations. It differentiates between four analytical layers: the context of roles, the context of functions, the context of structures and the context of norms. First, the context of roles refers to the stakeholders and beneficiaries in social innovation, their socio-demographic characteristics and their roles. This includes attitudes, skills, socialization, motivation and self-concepts, among others. Second, the context of functions encompasses the management and models of procedures, governance and business. This layer puts emphasis on the interlinkage and collaboration between actors and related network phenomena. Third, the context of structures layer depicts constraints and dependencies based on existing structures, such as institutions, political, economic or technological priorities. The fourth layer consists of the context of norms, i.e., the framework conditions and challenges posed by society. These are based on historical developments, the legal framework, ethical and professional standards and any other socially accepted standards that provide the basis for social innovation to occur.

Three levels of analysis and levels at which social innovation emerges are proposed by Cajaiba-Santana (2014), who differentiates between intra-social group innovations, inter-social group innovation and extra-group social innovations. The intra-social group innovations refer to basic values, beliefs, norms and conventions in a social group. The inter-social group innovations are based on various social groups that have a competitive or collaborative relationship, or both. The third level of extra-group social innovations is the macro-level of social systems.

The list in Table 1 is not exhaustive and combines different aspects of social innovation. Nevertheless, it shows different criteria according to which translation in citizen science can be analyzed regarding its contribution to social innovation. Since not all these dimensions, layers, models, and criteria can be considered in this study, the focus of the discussion will be on agency, institutions, and social systems (Cajaiba-Santana, 2014) as well as four key elements of social innovation, i.e., satisfaction of a need, innovation of the solution, change of social structures and relationships as well as the increase in society’s capacity to act (Portales, 2019).

### Zooniverse

Zooniverse is a citizen science platform, which was launched in 2007 inviting members of the public to engage in its first project, namely Galaxy Zoo, in which volunteers classified images of galaxies. Due to the success of Galaxy Zoo, the Zooniverse team (from the United Kingdom and the United States) engaged in new research areas (beyond astrophysics and also beyond natural sciences), participant tasks (classification, annotation, transcription, etc.) and user interfaces. Zooniverse projects have a focus on the analysis of large amounts of data that cannot be done by researchers on their own. Zooniverse users can analyze research data in the form of video, audio or images directly on the Zooniverse project pages after being instructed on how to conduct the relevant analysis. Researchers provide tutorials or guidelines that help participants identify, classify and label data according to the researchers’ requirements. Zooniverse is built on a domain model comprising the user, i.e., the volunteers participating in tasks; subjects, i.e., the elements that users are asked to annotate, transcribe or classify, such as light curves or museum specimen labels; workflows or tasks; classifications done by the volunteers; groups of subjects to allow for different displays on Zooniverse or different procedures of analysis; and, finally, a project, i.e., the individual citizen science project on the Zooniverse platform that is associated with subjects, classification and groups (Simpson et al., 2014). In 2019, approximately two million people had engaged in more than 150 Zooniverse projects to support hundreds of professional researchers in a variety of academic disciplines, ranging from physics and astronomy, climate science, ecology, biomedical research to the humanities. The volunteers’ contributions in the form of tagging, marking or transcribing of images, videos and audio on the Zooniverse platform resulted in more than 160 peer-reviewed publications (The Zooniverse Team, 2019).

Zooniverse allows for the translation of individual Zooniverse project pages. Researchers can request a translation of their material on Zooniverse from (volunteer) translators all over the world (TTFNROB, 2014) to increase the number of participants contributing to their project.

The Zooniverse translation platform was also discussed as a tool for translator education, to familiarize translation students with translation platforms, their interfaces and workflows, including quality assurance (Michalak, 2015) and produce translations that are actually needed and publicly accessible.

Zooniverse was selected for the analysis because it offers translations directly on the website (compared to other citizen science platforms, where basic information about the project is provided only in English, and users can obtain further information by clicking the link to the actual project website). In comparison to other citizen science directories or project platforms, Zooniverse allows users to directly work on the Zooniverse platform without being directed to the project website or having to work outside Zooniverse. Zooniverse is therefore an interesting research object for translation scholars, allowing for translation flow analysis and social media analysis (Desjardins, 2021). 

When the data for this study were extracted from the Zooniverse platform (in November 2020), 83 active projects were listed. However, only some of these projects offered content, i.e., information about their project or tutorials in English and (at least) one other language. Therefore, exclusion criteria were applied to the list of active projects on Zooniverse. Therefore, projects whose Zooniverse project page was only available in English were excluded. This reduced the final list to 17 projects. Another exclusion criterion would have been a regional focus of a citizen science project. This would be the case, for example, if a project in the field of biology or environmental sciences addresses only certain animal or plant species in a region. This regional focus would already exclude prospective participants outside this region. Thus, these projects would not address an international audience already by design and may not require translation. As assumed, this also held true for the projects on Zooniverse: Projects with a regional focus (in an English-speaking area), e.g., *Boston Phoenix, 1974!, London Bird Records, Nest Quest Go: Eastern Bluebirds, Notes from Nature: Plants of Arkansas, Snapshot Wisconsin or Scotus Notes: Behind the Scenes at Supreme Court Conference* were only available in English.

## Results

Translation receives considerable attention on Zooniverse. On the one hand, the Zooniverse website itself, i.e., the navigation and some contents are available in English and some other languages. Moreover, several citizen science projects listed on Zooniverse are available in more than one language. On the other hand, translation is also a topic discussed on the Zooniverse blog and in the general Zooniverse forums. On the Zooniverse Talk discussion forums, users recurrently ask for the translation of project pages or request translation features on Zooniverse. Zooniverse also provides a list of publications related to projects. Interestingly, this list only contains publications in English.

Since there is no translation policy, individual projects on Zooniverse decide on their own if their contents should be translated. However, the translations on Zooniverse are currently rather user-driven. “All translation effort comes from volunteers keen to bring the projects to their own communities” (Simpson, 2015). To become a volunteer translator for Zooniverse, users have to ask to be added as a translator to the project (indicating the target language). Then, they receive further instructions. If a volunteer translator wishes to contribute translations to a project on Zooniverse, the translation workflow itself therefore starts with contacting the project owner. Then, the project owner requests the Language option on Zooniverse, if not already enabled, and adds the volunteer as a Translator to the project. The project owner can select a language, in which the Translators work. The translations can then be published (and still be edited). This also means that if modifications are made in the English source text, these modifications are not simultaneously made in the target language. The comments on a project are not translated. Participants can however write their comments in any language, while it is recommended to GoogleTranslate them. The translators are primarily self-selected volunteers who are aware of Zooniverse or have already contributed to a Zooniverse project.

Interestingly, the topic of translation is mentioned on the Zooniverse blog predominantly in the year 2014. This was the year in which the Accessible Citizen Science for the Developing World project was gaining ground. In 2014, a call for volunteer translators on Zooniverse was issued. Within a short period of time, volunteer translators started to translate nine Zooniverse projects, starting in 11 languages. Especially volunteer translators from Spain and Germany were very active. The demand for translation was reflected by the usage figures. Between 2012 and 2015, the Zooniverse website traffic statistics showed that the percentage of users (who use a language other than English in their browser) visiting the English website decreased from about 65-70% in 2012 to 51% in early 2015. For individual translated project pages, this number was even 40% (Simpson, 2015). Since 2014, the topic of translation has not received much attention on the Zooniverse blog. Other research (Desjardins, 2021) also suggests that the translation features on Zooniverse are no longer further developed. However, in the Zooniverse forums, individual users are posing questions related to translation. A recent survey conducted to further develop the Zooniverse user interface revealed that the users still would like to have translations in more languages (Rother, 2018). In the discussion forums on Zooniverse, translation is a recurrent topic addressed by users. They are directing requests toward the Zooniverse team, e.g., introducing more multilingual Zooniverse features and more translation features. This demonstrates that the translation on Zooniverse is rather driven by the users, while no translation policy by Zooniverse is available (Desjardins, 2021).

The analysis of the Zooniverse projects showed that, from a total of 83 active projects, 66 projects were only available in English, while 17 projects were available in at least two languages (Table 2), always including English (while one project included only tutorials in other languages). This means that 20% of the Zooniverse project pages were also available in another language in addition to English. The most frequent language was, of course, English, followed by French (11 projects), Spanish and German (7 projects each). Other languages that were also represented on Zooniverse project pages include Italian, Czech, Dutch, Swedish, Portuguese, Polish, Japanese, Chinese, Ukrainian, Arabic, and Hebrew. Fourteen of the seventeen projects were projects in the field of natural sciences while the remaining three projects can be assigned to the humanities.

**TABLE 2:** Zooniverse project pages available in at least two languages.

Title of the Zooniverse project	Languages	Number of languages
Every Name Counts	EN, DE	2
NestCams	EN, DE	2
Invader ID	EN, ES	2
American WWI Burial Cards	EN, FR	2
Beluga Bits	EN, FR	2
Galaxy Zoo: Clump Scout	EN, FR	2
Snapshot Hoge Veluwe	EN, NL	2
Plant Letters	EN, PT	2
Scribes of the Cairo Geniza	EN, AR, HE	3
Galaxy Zoo	EN, FR, ZH	3
Radio Meteor Zoo	EN, FR, ES, NL	4
Iuganas from Above	EN, Tutorials: DE, ES, FR	4
Penguin Watch	EN, CS, ES, FR, ZH	5
Chimp&See	EN, DE, ES, IT, FR, CS	6
Radio Galaxy Zoo: LOFAR	EN, DE, IT, PL, NL, SV, FR	7
Backyard Worlds: Planet 9	EN, FR, ES, IT, PL, PT, DE, JA	8
Disk Detective	EN, ES, FR, IT, PT, PL, DE, UK, JA	9

The majority of the 17 projects that also included a language other than English were bilingual (8 projects), trilingual (2 projects) or offered information in four languages (2 projects). One project each supported six, seven (*Radio Galaxy Zoo: LOFAR*), eight (*Backyard Worlds: Planet 9*), and nine (*Disk Detective*) languages.

In addition, some Zooniverse projects also had a project website outside of the Zooniverse domain. This website or parts thereof were also available in other languages not used on Zooniverse, such as *Athena* (English and Dutch) or *Taranaki Mounga* (English and Maori). However, these projects were not included in the analysis since the focus was on the availability of other language versions on Zooniverse only.

From the point of view of localization, none of the citizen science project pages that were available in at least two languages, were localized, i.e., adapted to the relevant locale. First, locales or language varieties were not taken into account. The translations provided were in “French” or “Spanish”, but the locale was not specified, such as French of France, Canadian French, etc. The same also holds true for Spanish, e.g., Spanish of Spain or Spanish in the Americas, as a rough differentiation. Second, the look and feel of the Zooniverse citizen science project pages were the same for all languages. This means that neither the colors, the navigation or the information flow, among others, were adapted to the relevant culture. Only the text on the pages was translated. However, localization may run counter to the principle of ensuring a common look and feel of the entire Zooniverse platform, and, thus, of all projects. Moreover, as mentioned before, localization already needs to be considered during website design. Interestingly, the *Scribes of the Cairo Geniza* project has a design that deviates from the other projects on the Zooniverse platform. In addition to English, this Zooniverse project page is also available in Hebrew and Arabic, i.e., languages that are usually written and read from right to left. Whether the translation into these languages necessitated the deviating web design or if there are other reasons, such as reasons related to the display of the material and the completion of the tasks, would need further investigation.

Another observation is the selection of languages in which the Zooniverse project pages were translated, showing a clear tendency of languages with a higher status or high resourced languages, such as German, French or Spanish in addition to English. Only two projects offered translations into Mandarin Chinese, even though this language has the highest number of first-language speakers, and thus, a large pool of potential participants. In some cases, the selection of the languages may also depend on the topic of the project itself, such as in the *Scribes of the Cairo Geniza* project, which invites volunteers to work on pre-modern and medieval Jewish texts that had been hidden in Cairo for centuries. Therefore, the use of Hebrew and Arabic may result from the research objects themselves.

From the perspective of terminology, the analysis of the Zooniverse projects showed that the use of domain-specific terminology was reduced to a minimum. If domain-specific terminology was used, it was either explained directly in the text where it occurred or additional information was provided, such as in the FAQ or directly in the text. The project *Disk Detective*, for example, used the FAQ to briefly explain acronyms or proper names of tools and resources. The project *Scribes of the Cairo Geniza* explained terms directly in the text, e.g., the term “geniza”. The number of domain-specific terms was rather low, which made the texts comprehensible to a non-specialist audience.

## Discussion

In the following, these results are related to cultural differences that are negotiated through translation as well as translation validation in the case of crowdsourced translation for citizen science projects. Furthermore, they are embedded in a broader context based on the following basic criteria of social innovation: agents, structures and social change. The latter is discussed from the perspective of the transformative potential of translation in and for citizen science.

### Crowdsourced Translation

The translations on Zooniverse are a combination of both solicited and unsolicited translations (Jiménez-Crespo, 2013). Solicited translations, i.e., an organization issues a translation call to a community, could be predominantly observed in 2014, connected to a project aimed at increasing the accessibility of Zooniverse. Today, Zooniverse translations are rather non-solicited since self-selected users organize and complete translation tasks themselves without being requested to do so. However, Zooniverse provides the related infrastructure, i.e., a translation platform.

Zooniverse is thus also an example of crowdsourced translation where “translation consumers are increasingly becoming translation producers” (Cronin, 2010). This has also far-reaching implications for translation theory, which has been characterized by production-oriented models. These models assume that an agent produces translations that are consumed by an audience. Crowdsourced translation, however, means that the actual audience produces the translation on its own. Thus, they are no longer unknowable recipients of translation, but they are becoming active translation agents, i.e., producers or prosumers (Cronin, 2010). Moreover, since plural points of view and various modes of interaction are taken into account, the translation process and translation decisions can become more transparent (in comparison to translations done by professional translators). However, in crowdsourced translation endeavors quality assurance does usually not follow the principles found in the translation industry. In the translation industry, for example, the standard ISO EN 17100 “Translation services–Requirements for translation services” (ISO, 2015) specifies that translations are produced by professional translators (having a relevant university degree and/or experience) and that a second bilingual person revises the translated version (four-eye principle), among others. Moreover, in the translation industry quality evaluation and estimation metrics are heavily applied, such as BLEU for machine translation, the Multidimensional Quality Metrics (MQM), the quality assurance model by the former Localization and Internationalization Association (LISA QA Model) or SAE-J2450, which was initially developed for the automotive industry, but is also applied in other contexts. While these quality metrics enjoy popularity in the translation industry, these are not very common in the field of crowdsourced translation, including the Zooniverse translation platform. Reasons for this might be that their use requires some training and experience. Additionally, inter-annotator agreement has to be considered when evaluating the quality of translations with these metrics.

This shows that we can draw interesting parallels between crowdsourced translation and citizen science. Crowdsourced translation (and citizen science alike) require project and community management. Three important steps when crowdsourcing translation can be identified: a plan for crowdsourced translation, community building and support as well as the creation of a collaboration platform (Dunne and Dunne, 2011). When starting a platform for crowdsourcing translations (similar to citizen science) it is crucial to identify the community and to know their motivations to meet their expectations. Zooniverse already identified their users’ need to contribute to the translation of the platform itself and of individual project pages and provided a translation platform.

According to the literature, the recommended look and feel of a collaboration platform include a landing page providing an overview of projects, allowing for account management, informing about the terms and conditions, tasks and roles and facilitating the recognition of members, etc. After registration, the landing page should provide an overview of project management and the user role, such as translator, the tasks assigned, monitoring, and the type and volume of the items to be localized. To collaborate successfully on a crowdsourced translation project, collaboration needs to be supported with shared workspaces and resources, such as terminology management or glossaries, features for revision or voting on translations, style guides, a chat function or testing of localized versions. Moreover, the user interface for the actual translation should allow for terminology search, translation memory lookup and review. It is important that the volunteer translators do not have to handle code, but just the content of the page which needs to be localized (Dunne and Dunne, 2011).

Although the translations on Zooniverse are strongly user-driven, Zooniverse may also prepare a translation policy, appoint a project manager for the crowdsourcing of translations and define individual goals, such as increasing linguistic diversity on the platform. Another parallel between crowdsourced translation and citizen science is that users have to receive clear task descriptions, such as which contents to translate, how to manage terminology and how to ensure quality (e.g., consistency, accuracy). Moreover, the ownership of the results should be clarified in advance, e.g., if the translators have to agree to any terms and conditions. Additionally, crowdsourced translation requires constant community management, and thus communication (about task distribution, feedback on translations, quality evaluation, process management, acknowledging the contributions of the volunteers, etc.). Community management necessitates transparency, building of trust, opportunities for mentoring so that expert users can help novice users. Moreover, it requires a clear definition of roles and processes to manage the expectations of all persons involved. In the case of Zooniverse, the volunteers involved in the translation project receive support from the organization, either from the Zooniverse team or the leaders of individual citizen science projects on Zooniverse. Moreover, documentation on how to use the translation platform would benefit the users, e.g., how translation processes are defined and how quality is assured. Depending on the motivation of the users, the project managers can also acknowledge and recognize the contributions of the volunteer translators, either by issuing certificates, providing opportunities for learning or competence development, highlighting individual contributions, using leader boards, etc. Regarding the recruitment of volunteer translators, Zooniverse already has a community (of citizen scientists) of which some members are interested in producing translations for Zooniverse as well. Another option to recruit volunteer translators would be to extend the reach to potential volunteer translators who are not already familiar with Zooniverse.

The accuracy of the translations on Zooniverse has an impact on the quality of the tasks completed by the citizen scientists (if they rely on the translated instructions). The translations have to ensure that the participants in citizen science projects who use the translated version of a project’s page fulfill the tasks as intended by the researchers. To ensure that the source and the target text have the same meaning, an option would be to involve professional translators for quality evaluation, consistency checks regarding terminology (or style guide, if any) and integration with the source content.

Another option is multiple revision steps or community voting. The quality of crowdsourced translations may thus be assured through peer review, such as voting systems to decide on the “best” translation (Jiménez-Crespo, 2013). A third, more extensive option would be the validation of translations as applied for research instruments used in cross-cultural research endeavors.

### Validation of Translations

In the translation industry, the aforementioned quality metrics are applied to ensure the quality of translation, including accuracy, fluency, terminology, style, locale convention, etc. [in MQM (Lommel et al., 2014) terms]. Another approach (translation validation) can be found in cross-cultural research. Here, the translation of research instruments, such as questionnaires, undergoes several evaluation and validation steps to ensure cultural adaptation and equivalence in the target culture. Generally, translation validation requires various steps of instrument translation, cultural adaptation, content validation and equivalence assessment. These are important to ensure that the meaning of the items, the dimension integrity and validity are constant across cultures. Different equivalence criteria for cross-cultural research with instruments have been proposed. These criteria are, for example, content equivalence, semantic equivalence, technical equivalence, criterion equivalence and conceptual equivalence. These should ensure that the contents of the items are relevant to the aspects of each culture that are studied, that the meaning of the items is the same, that the method of assessment is comparable in the cultures, that the interpretation of the variable measurement are the same according to the norm in each culture and that the instrument measures the same (theoretical) construct in each culture. To ensure instrument equivalence, both qualitative and quantitative methods can be used and combined, including forward and reverse translations, expert evaluation, feedback questionnaires, pilot testing, participant review or cognitive interviews. According to this, the translation validation can consist of three phases and seven steps. The first phase, the instrument translation encompasses the steps: 1) instrument review and translator selection, 2) forward translations with synthesis, 3) reverse translation with reconciliation. The second phase, cultural adaptation, comprises 4) pre-test, 5) cognitive interviews, 6) research team review with item revision. The third phase, content validation and equivalence evaluation, consists of 7a) subject-matter expert evaluation and content validity as well as 7b) subject-matter expert evaluation of equivalence (Palmieri et al., 2020). The result should be an instrument in the target language that “asks the same questions, in the same manner, with the same intended meaning, as the source instrument” (Palmieri et al., 2020).

In different rounds, the cultural adaptability, the clarity of the translation, the cultural relevance and readability are assessed by different agents in the process. The cognitive probing step has a strong focus on terminology. Especially terminology may cause difficulties because it is embedded in a certain cultural system that relies on different categories, such as different terms for private and public hospitals (Palmieri et al., 2020).

Translation validation for cross-cultural research has a focus on equivalence, cultural applicability (adaptation) and cultural relevance, including readability and cultural adaptation for content validity. This should ensure that the instructions given to participants are interpreted in the same way in different cultures. In short, the translated instructions should not change the results. On Zooniverse, there is no translation validation according to the process described above. The translations on Zooniverse are not back translated and there is no systematic evaluation if the translations impart the same meaning as the source text. On Zooniverse, the translations can be revised after they have been published. As a result, users may see different instructions on translated project pages. These can be seen as reasons to question the accuracy and reliability of the translations since this may have a (negative) impact on the way how the participants complete tasks, and thus, jeopardize the research results. However, this has to be assessed in view of the fact that some Zooniverse users who contribute to a project that is only available in English reported that they translate the Zooniverse project pages and instructions with freely available machine translation systems into their language. This shows that researchers asking volunteers to complete citizen science tasks on Zooniverse cannot control how participants who do not use English as a preferred language interpret the task and if they use machine translation systems to understand the instructions. Since the quality of translation of freely available machine translation systems differs significantly between language pairs and domains, it cannot be guaranteed that the machine-translated text imparts the same meaning as the source text and that the machine-translated instructions are accurate enough so that the participants fulfill the task correctly (as intended by the researchers).

Although the translations on Zooniverse are not validated (according to the principles presented above) and can just be revised, they are nevertheless an important step of social innovation in citizen science. This echoes social innovation literature that states that grassroots innovations (in contrast to top-down innovations) that provide bottom-up solutions and offer strategies for cultural change can effectively respond to local concerns and contexts (Grimm et al., 2013).

While translations done by volunteers are sometimes criticized for not being as accurate as those of professional translators, volunteer translators usually take their tasks seriously. Furthermore, the quality of the translations is sometimes thoroughly evaluated by the community itself. Moreover, in forums, on advice pages or through mentoring, volunteer translators provide guidance and support to others. “These volunteers, unlike their professional counterparts, are actively encouraged to account for cultural distance and to intervene on the text, and are fostering communication across language and cultural divides” (Katan, 2016). Therefore, translations done by volunteers who are also participants in these citizen science projects would lead to a more accurate translation (compared to machine translation) since the volunteer translators have already participated in the project and are aware of the tasks themselves. In accordance with other crowdsourced translation activities, in which the users are translating the product or service themselves, the volunteer translator community on Zooniverse has already acquired profound knowledge of the project, its topics and tasks since they accumulated experience while completing the tasks themselves. This is in accordance with the literature that states that organizations engaging in crowdsourced translation with their users may draw on the knowledge and motivation present in their users. This helps increase the suitability and acceptability of their products and services or win the loyalty of customers (Massardo et al., 2016) (or participants in the case of citizen science).

While crowdsourced translation on Zooniverse has several benefits, it also raises issues of cultural differences and how far cultural differences can be bridged by translation.

### Cultural Differences

Different cultures do not only experience and see the world in many different ways but also make sense differently. Cultures differ in many aspects, including differences in value judgements (ascribing different values to things), differences in existence of abstract things (e.g., using abstract nouns for non-physical things), differences in the existence of concrete things (multiple words for snow in some languages), differences in relationships between things, differences in reason and thinking and differences in seeing things. Also, abstract nouns vary significantly between cultures and, therefore, translations may not capture the meaning equivalently. In some cultures, certain concepts may not exist at all. Therefore, speakers of one language see different things and differentiate things differently (group them into other categories). Moreover, within a culture there are subcultures organized around lifestyle, age, geographical location, and work, etc. These subcultures, again, experience and make sense of the world differently. This means that values, abstract systems, forms of reasoning and logic may also vary considerably within a culture (McKee, 2003). Thus, translation means to negotiate cultural differences. Therefore, also obstacles can arise from translation. One of these obstacles is (culture-bound) terminology.

#### Terminology

Different schools of terminology exist. While traditional terminology defines terminology as the entirety of concepts and their designations in a specialized area (RaDT, 2017), socio-cognitive terminology starts from units of understanding (that are often characterized by their prototypical nature) that depend on human language (and understanding) (Temmerman, 2000). The main difference between these two paradigms is that the first is objectivist, while the latter is experientialist.

In the present study, terminology is defined according to an ISO standard. The main elements in terminology are concepts and terms within a domain. A domain is a sphere of knowledge, subject field or activity that has its own social context, specialized culture and linguistic characteristics (ISO, 2002). Examples of terminologies include tax law, ornithology or precision medicine. Terminology facilitates communication between specialists and plays a crucial role in knowledge transfer and knowledge management (also across languages). While concepts are mental representations of phenomena and objects (units of thought) within a specialized field or context (ISO, 2002), terms are the linguistic expression of concepts. For example, the term “mouse” may either refer to the concept of the animal (mouse) or the concept of the computer mouse.

Cultures may have different knowledge and thus different knowledge systems. Similar to translation (multilingual) terminology tries to find equivalences of concepts in other languages. However, there may be fundamental differences between cultures. This is also at the core of socio-cognitive terminology, which elaborates on the “interaction between the world, language, and the human mind” (Temmerman, 2017). Languages reflect how humans understand, perceive and conceptualize the world. Since human understanding and new concerns in society evolve over time, languages and thus also terminology change (Temmerman, 2017). If there is a paradigm shift or revolutionary change, the transition period is also characterized by concept changes and term changes (Kristiansen, 2014).

There are “different degrees of cross-linguistic equivalence” (Temmerman and van Campenhoudt, 2014). On the one hand, there may be cultural uniformity in some domains, such as in accounting, while in other domains, such as law, there is a clear culture boundness (Temmerman and van Campenhoudt, 2014). Therefore, not all concepts are difficult to transfer from one culture to another, but especially those that are strongly culture bound (Kristiansen, 2014). This culture boundness is based on the assumption that cultures shape the human brain. Therefore, human cognition differs between cultures, even between closely related cultures: “our modalities of experience and our perception cannot be separated from the environment where we live and our previously stored experiences” (Faber and León-Araúz, 2014).

#### Bias and Multilingualism

The topic of translation is rarely problematized in the English citizen science literature. It seems that many citizen science projects assume that the participants are proficient in English. According to a study by Desjardins, (2021), explicit or implicit Anglocentrism, including epistemologies and computer programming, is also an issue in citizen science. Moreover, (even) if there is translation, it usually enriches English-language scholarship, i.e., the translation flow (knowledge transfer) is directed from a language into English. With regard to online citizen science, practices and structures have been addressed as reinforcing Anglocentrism, which leads to inequities and asymmetries when exchanging (scholarly) knowledge and cultural capitals. However, the (potential) participants in citizen science projects are characterized by diversity, such as language, culture, and education, etc. A recent study analyzing the translations and localizations on Zooniverse demonstrated that in 2019, nine projects, all from the natural sciences, were translated, i.e., the Zooniverse project pages were available in at least two languages or had translation features. Since translation considerations are usually not taken into account when designing a citizen science project on Zooniverse, this may reinforce epistemological biases, which may lead to limitations for pluralism and diversity. This study came to the conclusion that there is “limited linguistic diversity and generally Anglocentric modes of knowledge creation and dissemination” (Desjardins, 2021). Authors affiliated with Zooniverse are aware of some of these biases as well: Zooniverse is biased toward English-speaking volunteers who use browsers with high-speed connections. This raises issues of accessibility and international participation. Therefore, the former project Accessible Citizen Science for the Developing World aimed at improving the Zooniverse translation tools and reaching more diverse participants (in addition to increasing the accessibility of the Zooniverse project builder) (Simpson, 2015).

Therefore, the question arises whether a citizen science platform launched in a multilingual environment, compared to Zooniverse, would consider multilingualism and translation already from scratch. Since the EU-Citizen.Science platform (eu-citizen.science) was launched in such a multilingual context, it may support different languages and translation features by design.

The EU-Citizen.Science platform (EU-Citizen Science, 2020) emphasizes that citizen science should become a means for the democratization of science. Its mission is to share knowledge across networks, including researchers, policymakers, participants in citizen science projects, practitioners and society in Europe. Since the platform serves as a knowledge hub and community hub in Europe, we may draw the conclusion that this knowledge is made accessible and exchangeable in different languages (also by means of translation). Especially, “Objective 3: Empower” emphasizes that a wide range of stakeholders can become citizen scientists, start and implement citizen science projects and approaches in a professional way. “Objective 4” addresses new ways of participatory governance and a stronger link between citizen science and policy, whereas “Objective 5” aims at citizen science becoming mainstream in public engagement, education and science communication. To reach these objectives, translation or localization are important, because science communication, education, the implementation of citizen science initiatives and policymaking in Europe are usually taking place in a language other than English.

Interestingly, EU-Citizen.Science (as at January 5, 2021) offers content predominantly in English, including announcements of news or events, forum discussions and blog posts. Among the 87 resources available on the platform, some are available in languages other than English. Among the 19 training resources (although the language filter is enabled) none are available in a language other than English. However, the teaser video introducing the EU-Citizen.Science platform is available in 12 languages. Although the overview of citizen science projects lists projects from all over Europe and beyond, the projects either bear English-only names or provide an English explanation of the original title in brackets. On the individual project pages on EU-Citizen.Science, 147 projects (as at January 5, 2021) are briefly described and tagged in English. The individual project websites themselves (not under the EU-Citizen.Science domain) also give information in languages other than English. Before drawing conclusions about Anglocentrism and an underrepresentation of multilingualism on EU-Citizen.Science, it is important to bear in mind that the EU-Citizen.Science platform was launched only recently. However, there was no information available whether it will feature and promote multilingualism and translation in the future.

The efforts made by Zooniverse and EU-Citizen.Science to increase linguistic diversity and knowledge exchange by means of translation deserves recognition. In contrast to other internationally visible citizen science platforms, Zooniverse and EU-Citizen.Science are supporting languages other than English and the localization of their content. This multilingualism and translation are ingredients for social innovation in citizen science.

### Translation and Social Innovation in Citizen Science

Social innovation can refer to both, the process and the outcome. Social innovation, thus, must not necessarily aim at a target. The process itself can be an innovation outcome as well. Through co-production, resource sharing and cooperation, new social relations may emerge between previously unrelated or uncooperating stakeholders. The social capital can increase by improving a society’s capacity to act and by helping create resilience and sustainability in societies (Grimm et al., 2013).

#### Agents

Social innovation is characterized by seeking social change and a focus on (societal) values, the promotion of cooperation among actors and the improvement of relationships. This is in contrast to economic innovations, where the commercial benefits and competition among actors are dominant (Portales, 2019). Social innovations often result in novel social relationships between organizations and individuals from different walks of life. The process of co-creating solutions to social needs can thus lead to transformative change (Grimm et al., 2013). This co-production of solutions means that actual users also make decisions, which is also the case on Zooniverse. However, these agents are also subject to different merit (and value) systems that may be conflicting. Therefore, the interplay between policy, cultural norms and individual capacity should be considered. This is also the reason why measuring social value can be problematic (Milley et al., 2018).

Social innovation requires participation and engagement from a wide diversity of actors, thereby requiring individual citizens to assume responsibility (Grimm et al., 2013). Citizens become active participants in collective decision-making. This responsibility is based on engagement and empowerment (Nicholls et al., 2015).

This is also the case for Zooniverse translations that are predominantly initiated proactively by the citizen scientists themselves who wish to bring the projects to their cultures. Moreover, social innovation is often spurred by individuals. They did not only voice a (social) need (which was also reflected by a Zooniverse survey (Rother, 2018)) but are also, to a certain extent, co-creators since they are directly involved in the development or further improvement. In the case of Zooniverse, this leads to a new relationship between the researchers, the Zooniverse staff and the citizens. However, the diversity and number of stakeholders that is usually required in social innovation processes is rather low. Different specializations, including competences and resources, need to be combined to arrive at social innovations. The driving forces, or agents, in social innovation processes have been grouped into different categories by different authors. One of these categorizations is the triad of public bodies (public), private companies (private) and NGOs/NPOs (civil society) who identify problems, provide resources and infrastructures that complement each other and create synergies (Nicholls et al., 2015; Butzin and Terstriep, 2018).

Other authors define individuals, organizations and social movements as key agents in social innovation processes (do Adro and Fernandes, 2020). Social innovation would, thus, require that every member of the community is actively involved in the innovation process. This is also reflected in funding schemes of the European Union that emphasize the role of citizens in research and innovation (processes). Since these citizens speak different languages, translation is an essential component of active participation. Only if members of the public understand the material given to them or what is expected from them, they can be agents of social innovation. Since many projects provide information primarily in English, they may exclude a large proportion of the agents required for instigating change. Therefore, translation can help remove the language barrier and any misunderstandings that may arise due to a lack of language proficiency if information is only provided in English. When highlighting the agents in social innovation, it is also important to address the inclusion or exclusion of certain agents in the process.

In citizen science, the main agents of social innovation would be the researchers, the participants, and in the case of translation, the translators (and platform developers). However, these agents are embedded in communities, organizations and institutions. Therefore, also research institutions, funding bodies, the professional and personal environment of a person, etc. play a role.

Furthermore, translation also plays an important role for Zooniverse, which states: “Our goal is to make it easy for anyone to contribute in a valuable and meaningful way to real academic research” (The Zooniverse Team, 2019). By overcoming language barriers through translation, it is easier for ‘anyone’ to contribute to academic research.

“One of the defining features of social innovation is that it provides insights and develops capacity and soft infrastructure (intangible assets such as know-how, intellectual property, social capital, etc.) that endure and can be utilized by other sectors and forms of innovation” (Grimm et al., 2013). According to this, the translation on Zooniverse helps develop capacities. The volunteer translators gather know-how on translation and the use of technology, i.e., the translation platform. They create their intellectual property through translation. Moreover, the code for the Zooniverse translation platform is available as open-source code on GitHub (https://github.com/zooniverse/Translator/), which makes it re-usable by others.

A peculiarity of the translation of citizen science project materials is the comprehensibility for a non-specialist audience. Usually, the authors of the source texts in citizen science projects already consider the necessity to re-phrase, generalize or simplify texts and avoid using terminology that the audience may not understand. Therefore, also the translation of these texts has to strike a balance between domain-specific knowledge and general comprehensibility, between the loyalty to the disciplinary discourse and the loyalty to the readers.

On the Zooniverse platform, researchers do not only ask participants to volunteer for research but also translators to voluntarily provide translations for their Zooniverse project pages. This means that researchers draw on the effort and resources of two (different, but partially overlapping) groups of volunteers who want to contribute to the generation of a greater common good (which is also a basis of social innovation). If these groups are considered agents in social innovation, they are part of the process of innovation generation and diffusion. This will be discussed in the next sections.

#### Structures

The structures relevant in the current analysis range from the research landscape in general and the citizen science landscape in particular, to social developments, legal requirements and various other framework conditions. Since the focus of this analysis is the role of translation in citizen science, the following section concentrates on the citizen science landscape.

While translation of citizen science projects and material can help foster social innovation, translation is also embedded in framework conditions (structures). These framework conditions are, among others, the citizen science landscape itself. There are numerous citizen science project platforms and manifold citizen science projects all over the world. Especially if citizen science projects aim at addressing a global audience, they basically compete for the same participants. However, if these citizen science projects address different topics and offer different tasks to prospective participants, they may serve different motivations and interests of the volunteers so that they would not compete for the same participants per se. To illustrate this with projects from the Zooniverse platform: Users may select a project to which they wish to contribute based on the topic of the project and the tasks they have to fulfill. If participants are rather interested in biology, they may choose a project where they can engage in an activity with animals or plants. Then, they may select between projects based on a species that is more appealing to them. Zooniverse offers several projects from natural sciences, and biology in particular. Users can choose if they want to address, e.g., biodiversity in general (e.g., *Taranaki Mounga*) or animals in general (e.g., *eMamma*l). Additionally, they may select from different species. If persons are interested in animals, they can choose between penguins (*Penguin Watch*), birds in general (*NestCams*), chimpanzees (*Chimp&See*), beluga whales (*Beluga Bits*), etc. Despite of the topic, they may also choose between different tasks. If they are interested in astronomy, they can select from various projects, e.g., *Astronomy Rewind, Aurora Zoo, Bursts from Space, Galaxy Zoo, Planet Four: Ridges, Planet Hunters Tess* or *Spiral Graph*. The volunteer tasks in these projects range from contributions to the creation of a database of astro-referenced old astronomy images and measuring the curvature of spiral arms in galaxies to searching for undiscovered worlds or discovering networks of polygonal ridges. Some of these tasks are certainly more appealing to a certain group than others leading to a self-selection of participants.

While the topics and the tasks of citizen science projects may be seen as “barriers” to some groups of potential volunteers, another obvious barrier on the Zooniverse platform is the language barrier. People who are not confident in using English, or using scientific terms in English, etc. may be rather attracted to citizen science projects offering a website, training material, publications, etc. in their preferred language.

In this case, translation can help overcome this language barrier and may attract users to the platform that would otherwise not visit the platform or would not pay any attention to a certain citizen science project (if information is only available in English). Therefore, citizen science projects offering their project materials and tools in a language other than, or in addition to English, may draw from a larger pool of potential project contributors. Therefore, they may have a competitive advantage over citizen science projects that provide information in English only. Similar to economic interests, when companies localize their products to maximize profit and reach currently unreached target markets, citizen science projects may increase the number of their participants, by offering translated or localized versions of their websites and their tools. Through this translation or localization, they may either gain a competitive edge over their “competitors”, i.e., other citizen science projects or increase the number of potential volunteers by reducing language barriers. However, social innovation is not so much about having a competitive edge but rather about satisfying a yet unsatisfied human need.

Another framework condition in the citizen science landscape is the financial support for both the launch of projects and the maintenance of projects in the long term. Moreover, the persons involved in citizen science require appropriate incentive systems, such as institutional recognition of citizen science activities as proposed with the social impact indicator in addition to awards, prizes or privileges for the persons involved in citizen science (Schäfer and Kieslinger, 2016). The emergence and further development of citizen science in the past years can also be attributed to the framework conditions found in academia increasingly characterized by reliance on external funding, research questions requiring large amounts of data and the need of research to address societal challenges, the latter being enshrined in the third mission paradigm of universities. Citizen science may also be seen as serving a societal need. It can help combat distrust in science among society, align research with real-world needs and societal challenges and contribute to the empowerment of citizens.

#### Social Change: The Transformative Potential of Translation in Citizen Science

This section sheds some light on the role of translation used in citizen science projects and the ways of how translation can foster social innovation through citizen science activities. As mentioned before, social innovation is embedded in a social structure and requires the interaction between a variety of actors. The social innovation process is an open process and social innovators are usually deviating from prevailing paths, rules, routines and models. Therefore, altered social practices drive transformative social change (Howaldt et al., 2016). While it is already established that citizen science itself is a means of departing from established routines, models and rules in academia – and, thus, a form of social innovation (Butkevičienė et al., 2021), this transformative potential of translation in and for citizen science is less obvious. One of the reasons for this is certainly the practice of translation itself which looks back on a long tradition. Therefore, from the innovation dimension perspective, translation itself is nothing new or innovative. However, as described above, translation can trigger change. An example of this is the notion of “citizen science”. The concept of citizen science, i.e., the act of engaging members of the public in conducting (certain steps in) academic research on their own, may not exist in certain languages, e.g., because citizen science is not practiced. On the other hand, if the concept of “citizen science” already exists, a language community may already use a term in the respective language for it. The third option would be that the concept of “citizen science” is currently being introduced. This usually means that there is no term in the relevant language available for the practice of “citizen science”. Then, there are two options. First, the English term “citizen science” can be incorporated as a loanword into the relevant language. For example, in Austria “Citizen Science” is also used in German (the only adaptation being the capitalization of the words in accordance with the writing of nouns in German). The second option, which was adopted in Germany, was a calque, i.e., a loan translation, resulting in, e.g., “Bürgerwissenschaft”, being a word-for-word translation of the term “citizen science”. On the one hand, this example shows that translation, in its broadest sense, can help change the mindset as well as established practices. On the other hand, it also demonstrates the importance of localization, and in this respect intralingual localization. Although German is the official language in both countries (Austria and Germany), different ways of introducing the concept of “citizen science” were chosen. Similar to social innovation, this is rooted in certain framework conditions. One of these framework conditions in this example is the use of Anglicisms in the German language, where the language community in Austria is more willing to borrow words and phrases from English compared to the language community in Germany. While the use of the calque “Bürgerwissenschaft” may result in a better comprehensibility of the term among the general public, the loanword “Citizen Science” on the other hand can emphasize the novelty of the concept while having only one meaning and being basically free of (unintended) connotations. However, sometimes the concept of the loanword, i.e., the meaning of “citizen science” may change when being introduced to another language. This can be detrimental when persons from the language community from which the concept “citizen science” originated and persons from the language community using the loanword with another meaning use “citizen science” in a conversation. While the term is the same, i.e., “citizen science”, the meaning (the concept behind it) is different, which may cause (serious) misunderstandings. To sum up, the introduction of concepts (for example, in the form of loanwords or calques) from one culture to another may introduce alternative social practices that are at the core of social innovation.

Turning now to the ability of translation to instigate this change based on the assumption that social innovation encompasses (profound) changes in complex systems, such as constructs, institutions, relations and behaviors (Antadze and Westley, 2012).

First, translation is a means for knowledge transmission. Although knowledge can take various forms and can be transmitted by different means, language is the primary means to transmit knowledge. Since language barriers can also become barriers to knowledge transmission, translation plays an important role when it comes to knowledge transmission between languages. Translation, thus, can give access to information, knowledge, products and services, practices, etc. as well as different ways of thinking and interpreting the world. These knowledge and social practices may instigate change in the receiving culture by introducing new elements in the target culture. Translation is thus not restricted to language and texts, but it is a social and cultural activity (Bachmann-Medick, 2013). This linguistic and cultural diversity is crucial to find solutions to societal challenges. Nevertheless, translation also is an act of negotiation and mediation between different cultures and requires interpretation from the translator.

Translation is a form of appreciation: an appreciation of the source text, on the one hand, and appreciation of the participants in citizen science projects, on the other. The appreciation of the source text means that the effort of translation alludes to the fact that the text has a certain value, and its content needs to be disseminated in other languages. Translation also means the appreciation of volunteers in citizen science projects, since it is a welcoming and appreciative act to receive information in a preferred language. Additionally, translation helps to overcome language barriers and misunderstandings that may arise if participants have to use a language in which they are not fluent, such as English on Zooniverse.

Second, translation is a driver of change. Translation has the power to bring about change. This can be either a turn for the better or a turn for the worse. While translation can be used to exert power, to manipulate people or to impose certain ideologies, it can also have a positive impact by enriching the receiving culture. This positive impact may range from empowerment, representation of minority groups to negotiations to resolve conflicts in a peaceful way, new interpretations and the acknowledgment of diversity (Tymoczko and Gentzler, 2002).

Third, “translation” is a term that is also used beyond its own discipline, namely beyond translation studies. Translation is either used as an analogy or as a means of argumentation by scholars from other disciplines. Translation, thus, has an impact on theories and discourse beyond its discipline (Woodsworth, 2013). This translational turn in various academic fields has led to the enrichment of these fields with findings, methodology and approaches from translation (studies). Translational approaches consider contexts, cultures, differences, mediation, and connections, etc. and help negotiate differences, assess misunderstandings and show power asymmetries (Bachmann-Medick, 2013).

Fourth, translation helps to overcome language barriers (and cultural barriers). As the definition of translation already suggests, it aims at enabling communication between languages and cultures. Mutual understanding across languages and cultures is therefore key.

Fifth, translation also means adaptation (also referred to as localization). According to the functionalist theories in translation studies, translation has to fulfill a purpose and this purpose influences the translation strategies and the final outcome. This is closely related to localization studies. Localization studies differentiate between globalization, internationalization, localization, and translation. In localization studies, localization is defined as the adaptation of a product (or service) to a target market, i.e., a locale. This means that a product’s content, functions and look and feel are adapted to the requirements of the target market. In comparison to translation (according to localization studies), localization encompasses the adaptation of linguistic and non-linguistic elements, while translation only focusses on linguistic aspects of a product (or service) (Drewer and Ziegler, 2014). Thus, a well-localized product does not only meet the (cultural) expectations and preferences of the target audience (i.e., the content is culturally sensitive) but also the (legal and technical) requirements of the target market (Dunne and Dunne, 2011). This is briefly illustrated with a website of a citizen science project. Localization thus means that not only the text on the website is adapted (translated) but also the images, colors, functionalities, the navigation bar or the sequence or flow of information, symbols or fonts so that the localized website has the look and feel of an “original”, i.e., a locale-specific website. In certain cases, it may be even necessary to change the name of the project if the name of the project evokes undesired connotations in the target locale. This demonstrates the importance of cultural embeddedness and culture-bound terminology.

A special form of localization is intralingual localization, i.e., the localization between different varieties of the same language. Typical examples for locales are American English, British English, South African English, etc. Here, the characteristics and conventions of the target audience, i.e., speakers of the same language who use a different variety within this language, are taken into account.

Referring back to Zooniverse and the differentiation between internationalization, localization and translation. While localization creates a culturally sensitive product that meets the requirements of the target locale, internationalization lays the foundation for successful localization. In localization studies, internationalization refers to the design of a product that allows for the adaptation to the target market, i.e., allows for localization. For a citizen science project platform, this means that already in the website development stage, localization aspects are considered, such as bidirectional reading, change of colors, support of other fonts and character sets, e.g., Asian script, audio and video output, other units of measurement, such as date and time, decimal separators, symbols and text expansion for the translation into languages that are usually longer than the source text.

Sixth, when used as a metaphor or analogy, translation in the context of citizen science can also mean that citizen science itself is a translation (exercise) and a means to introduce and translate knowledge or academic principles to non-professional researchers as well as a means to introduce and translate social innovation.

Social innovation is characterized by the interrelatedness of social and institutional structures and agency (Cajaiba-Santana, 2014). Translation can permeate all these agents and structures and support their role in the process of social change as part of social innovations. This is also supported by the definition of social innovation by the Center de Recherche sur les Innovations Socials (CRISES) as cited in Agostini et al. (2017): Social innovation is “a process initiated by social actors to respond to a desire, a need, to find a solution or to seize an opportunity of action to change social relations, to transform a frame or propose new cultural orientations to improve the quality and community living conditions”. Translation has the potential to transform (cultural) orientations since it enables the communication between cultures.

When considering the various criteria derived from the social innovation literature, with a focus on the four key elements: “satisfaction of a need, innovation of the solution, change of social structures and relationships, and the increase of society’s capacity to act” (Portales, 2019), the (crowdsourced) translation of citizen science project information and websites satisfies a need, i.e., the need of volunteers who want to access products, services and information in their preferred language. It is important to bear in mind that the selection of the languages in which a citizen science project website is translated (or preferably localized) should be in line with the objective of the project. Often, the selection of the languages reflects their social capital. Regarding the “innovation of the solution”, translation is not innovative per se since it looks back on a long tradition and has been used in various contexts. Nevertheless, translation in and for citizen science can change social structures and relationships and help increase a society’s capacity to act.

On the question of the dimensions of social innovation (Moulaert et al., 2005) in relation to translation in citizen science, we may differentiate between the content (How does translation in citizen science satisfy social needs?), the process (How does translation change social relations in citizen science?), and empowerment (How does translation increase capabilities and access to resources?).

Social innovation (and partly also translation) is usually considered as an improvement or a positive change. However, innovations (and also translations) may have unintended consequences or externalities. While some stakeholders may benefit from social innovation, others may lose. Reasons for this can be the exclusion of some groups from the process, hijacking by extreme groups, the balance between financial and social objectives of social innovations and the risk and potential failure of social innovations. Moreover, to achieve real systemic change, dominant cognitive frames have to be overcome. This changing of cognitive frames as well as the challenging of normative roles and responsibilities may encounter resistance (Nicholls et al., 2015). This holds also true for the (crowdsourced) translations on Zooniverse that challenge the existing roles of professional translators and the decision-making responsibilities of researchers and platform providers. Translation changes social relations since it extends linguistic diversity, multilingualism and access and contributions to academic research. Volunteers wishing to promote or contribute to a project in their language now request (and produce) translations. From the perspective of the process dimension, this has an influence on governance and increases the level of participation. Moreover, it introduces some diversity to a platform that was initially created from an Anglocentric point of view and takes account of the fact that not every citizen scientist will be able to cope with English.

From a content perspective, translation fulfills a (social) need illustrated by the mainly non-solicited crowdsourced translations on Zooniverse, the translation requests by users and the use of machine translation for project pages by users. Although translation is accompanied by cultural differences, including terminological gaps, differences in seeing the world and in communicating with each other, which may be problematic in terms of unintended outcomes and misunderstandings, it can change social relations and increase empowerment. Translation is thus giving access to information, knowledge and resources. Translation increases capabilities since volunteers translate the content on their own. They get some control over the citizen science project as well as, the availability of information and resources in their language(s).

Especially technological progress and shifts toward the democratization of science can instigate (radical) change, also with regard to democratic knowledge creation and knowledge transfer. Therefore, citizen science plays a crucial role in knowledge translation (Heinisch, 2021). Participants in citizen science projects contribute actively to scholarly research and also dissemination, whereas translation is an important means of knowledge transfer. Thus, it can also serve as an instigator of social change and social innovation and can help deal with (epistemological) asymmetries (to a certain extent) by challenging existing paradigms and practices. To be successful, the citizen science community may need good practice guidance and policies on fostering linguistic diversity and translation (Desjardins, 2021).

Both crowdsourced translation and citizen science benefit from technological advances and the Internet as well as other developments, such as user-centered design, collaborative platforms, networks and user-generated content (shifting the focus away from mere passive users to active content producers) (O'Hagan, 2016).

Together these results provide important insights into the translation aspect of citizen science projects listed on Zooniverse. They demonstrate that especially the adaptation of material to a certain locale may indicate sensitivity to cultural differences and may be a driver of (social) innovation in and through citizen science. Although translation with regard to citizen science activities can result in the satisfaction of a social need and new social practices in the target locale that may increase the people’s capacity to act, it may also result in the perception that local knowledge and local traditions are disregarded. Therefore, the results further support the idea of translation being an act of negotiating linguistic and cultural difference. Interestingly, in citizen science, terminology needs to be translated not only into another language but also translated (in the figurative sense) to a non-specialist audience. While this study did not further investigate on the importance of translation in citizen science, it did partially substantiate an unequal distribution of symbolic capital among languages. This can be derived from the languages in which the case study projects were translated. This further supports the idea of symbolically dominating languages when translating material for citizen science projects. This asymmetry can allude to the fact that the actors involved in citizen science may not be equal which is an obstacle to social innovation in and through citizen science. Nevertheless, the results also demonstrate that translation in citizen science may lead to a change of practices, including new ways how academics interact with the participants in citizen science projects. These are ingredients for social innovations.

Terminology is at the core of academic disciplines. It aims at unambiguous communication among domain-specific experts. Due to its specialized nature, though, the use of terminology also means to exclude members of the public from academic discourse. This becomes especially apparent in citizen science. Here, scholars and members of the public are the main actors that work toward a common goal specified by the relevant citizen science project. To achieve efficient communication, both actors have to make concessions to each other from a terminological point of view. The “translation” of concepts from one culture to another may result in changed social practices that are the drivers of social innovation.

### Limitations

A limitation of this study is the concentration on the Zooniverse platform since it is primarily aimed at an international (English-speaking) audience. Moreover, the majority of the projects on Zooniverse have a focus on data analysis (and not on data collection). As mentioned in the discussion about agents and structures of translation in citizen science, the citizen science projects on Zooniverse may compete for the same participants, especially if they address the same topic and offer similar tasks. Since many Zooniverse projects ask volunteers to analyze data, persons interested in collecting data, e.g., being in nature and collecting samples or recording observations, etc. may not be attracted by the platform itself, since the tasks of Zooniverse projects can be basically completed from home by using only a computer.

The major limitation of this study is the focus on case studies on only one citizen science project platform. Therefore, further work in the form of in-depth studies of citizen science projects are needed to fully understand the implications of translation (and terminology) in citizen science and its effects on social innovation. Moreover, the interrelations between crowdsourced translation, multilingual project communication and citizen science beyond citizen science project platforms, including project websites and social media, would necessitate further analysis to take account of the multiple forms of translation in citizen science in the digital realm. Nevertheless, this work has been one of the first attempts to examine the relation between translation, citizen science and social innovation.

To develop a full picture of the role of translation, additional studies will be needed that further explore social innovation as a process. For the evaluation of social innovation as a process, developmental evaluation (Patton, 2016) may help to take account of the constant negotiation of a problem and its solution and the iterative emergence of a solution. Moreover, developmental evaluation is sensitive to the context and adaptation, i.e., the impact of innovations should be measured in their context (Antadze and Westley, 2012). Moreover, the situatedness of translation needs further investigation, especially the cultural and sub-cultural norms in different regions (Grimm et al., 2013), such as the consideration of different locales and also the cultural differences within locales.

Some citizen science projects on Zooniverse also have a project website outside the Zooniverse domain and among them are several projects that offer information on their website in other languages, i.e., languages that are not used on their Zooniverse project pages. Further research may investigate the reasons for not integrating these translations into the Zooniverse platform.

## Conclusion

To reach participants, citizen science projects need to speak their participants’ language. Especially for citizen science projects that have a global reach, translation is crucial to overcome language and cultural barriers to reach members of the public. Translation, understood as the transfer of meaning (of a text) from one language into another language, is essential for the transmission of information, knowledge and (social) innovations characterized by social change.

Although the translation of citizen science project websites and related materials and tools may contribute to social innovation, translation may not be primarily aimed at achieving social change but rather at reaching a broader audience. However, also the process (of translation) can lead to change, for example to new social relations and empowerment in and through citizen science. Languages offer different perspectives on the world. Seeing the world through a different lens (also through translation) is an important driver of social innovation. Translation may also result in an increased awareness for and appreciation of linguistic diversity in citizen science, the reduction of (cultural) biases and the consideration of translation by design.

Social innovation is characterized by the interrelatedness of social and institutional structure and agency. Translation can permeate all these agents and structures and support their role in the process of social change as part of social innovation. Thus, translation can contribute to the change of social practices and to the spread of social innovation, such as citizen science (as a manifestation of social innovation).

## Data Availability

The original contributions presented in the study are included in the article/Supplementary material, further inquiries can be directed to the corresponding author.
