# Corrigendum: The Role of Translation in Citizen Science to Foster Social Innovation

**DOI:** 10.3389/fsoc.2021.764339

**Published:** 2021-09-20

**Authors:** Barbara Heinisch

**Affiliations:** Centre for Translation Studies, University of Vienna, Vienna, Austria

**Keywords:** translation, localization, adaptation, social change, terminology

In the original article, we missed to cite Desjardins (2021) in the **Methods and Materials** section. The citation and additional text have now been inserted in **Methods and Materials, Zooniverse, paragraph 4**. The corrected version now reads:

“Zooniverse was selected for the analysis because it offers translations directly on the website (compared to other citizen science platforms, where basic information about the project is provided only in English, and users can obtain further information by clicking the link to the actual project website). In comparison to other citizen science directories or project platforms, Zooniverse allows users to directly work on the Zooniverse platform without being directed to the project website or having to work outside Zooniverse. Zooniverse is therefore an interesting research object for translation scholars, allowing for translation flow analysis and social media analysis (Desjardins, 2021).”

In the original article, there was an error. The reference for Desjardins (2021) was given in the middle of the paragraph, instead of the beginning of the paragraph. A correction has been made to **Cultural Differences, Bias and Multilingualism, paragraph 1**:

“The topic of translation is rarely problematized in the English citizen science literature. It seems that many citizen science projects assume that the participants are proficient in English. According to a study by Desjardins (2021), explicit or implicit Anglocentrism, including epistemologies and computer programming, is also an issue in citizen science. Moreover, (even) if there is translation, it usually enriches English-language scholarship, i.e., the translation flow (knowledge transfer) is directed from a language into English. With regard to online citizen science, practices and structures have been addressed as reinforcing Anglocentrism, which leads to inequities and asymmetries when exchanging (scholarly) knowledge and cultural capitals. However, the (potential) participants in citizen science projects are characterized by diversity, such as language, culture, and education, etc. A recent study analyzing the translations and localizations on Zooniverse demonstrated that in 2019, nine projects, all from the natural sciences, were translated, i.e., the Zooniverse project pages were available in at least two languages or had translation features. Since translation considerations are usually not taken into account when designing a citizen science project on Zooniverse, this may reinforce epistemological biases, which may lead to limitations for pluralism and diversity. This study came to the conclusion that there is “limited linguistic diversity and generally Anglocentric modes of knowledge creation and dissemination” (Desjardins, 2021). Authors affiliated with Zooniverse are aware of some of these biases as well: Zooniverse is biased toward English-speaking volunteers who use browsers with high-speed connections. This raises issues of accessibility and international participation. Therefore, the former project Accessible Citizen Science for the Developing World aimed at improving the Zooniverse translation tools and reaching more diverse participants (in addition to increasing the accessibility of the Zooniverse project builder) (Simpson, 2015).”

The author apologizes for these errors and states that this does not change the scientific conclusions of the article in any way. The original article has been updated.

